# Editorial: Updates on ulcerative colitis and Crohn's disease: from bench to bedside

**DOI:** 10.3389/fmed.2023.1285660

**Published:** 2023-09-14

**Authors:** Vito D'Andrea, Gaetano Gallo

**Affiliations:** Department of Surgery, Sapienza University of Rome, Rome, Italy

**Keywords:** inflammatory bowel diseases, Crohn's disease (CD), ulcerative colitis (UC), inflammation, computed tomography enterography (CTE), biomarkers, machine learning

Inflammatory bowel diseases (IBD) are lifelong and bothersome diseases, with a relapsing-remitting course, including Crohn's Disease (CD) and Ulcerative Colitis (UC) (1, 2). In the Western World the prevalence of IBD is higher and nearly 1% of people will be affected with IBD by 2030.

CD is a disabling, destructive and progressive disease in which the inflammation is transmural, commonly affecting the small bowel and potentially involving any portion of the gastrointestinal tract (3). Within 10 years of diagnosis, penetrating or structuring complications lead 50% of patients to surgery and post-operative recurrence is common (4). In UC inflammation is more superficial affecting the mucosa, confined to rectum or the sigmoid colon (distal colitis) in 30–50% of patients and characterized by bloody diarrhea combined with urgency and tenesmus. Within 10 years of diagnosis, 15% of patients need surgery and the risk of surgery is decreasing (5).

This Research Topic of Frontiers in Medicine, composed by six original articles on IBD, addresses several topics including non-surgical solutions, diagnostic aspects, translational research, and specific scenarios.

C-X-C motif chemokine ligand 12 (CXCL12) and its receptor C-X-C motif chemokine receptor 4 (CXCR4) have emerged as promising therapeutic approaches targeting tumor growth and metastasis (6). In this context, AMD3100 (Plerixafor) is currently the only FDA-approved small molecule CXCR4 antagonist and is the most frequently used drug targeting the CXCL12-CXCR4 axis in clinical trials (7).

Wu et al. showed that AMD3100 treatment reduced the inflammatory damages in the colonic mucosa ameliorating colitis-associated colon cancer development in experimental mice. AMD3100 regulated the functional phenotypes of peritoneal macrophages, including phagocytosis activity, recruitment, polarization, and the serum levels of cytokines such as IL-12 and IL-23.

Yang et al. showed that endoscopic indices combined with computed tomography enterography examinations (CTE) and clinical symptoms are of great value in the differentiation of CD and intestinal Bechet Disease. In particular, the authors considered three different scoring models, i.e., endoscopic features alone (model 1), endoscopic features combined with clinical symptoms (model 2), and endoscopic features combined with clinical symptoms and CTE (model 3), demonstrating that clinical symptoms and CTE improve the discriminatory power of endoscopy with the highest accuracy rate of 84.15%. For hospitals without the ability to perform CTE, clinical symptoms can also increase the differential ability of endoscopic features.

Li, Yan et al. showed that the use of the machine learning model containing multiple clinical and laboratory variables can serve as an effective non-invasive approach to predicting endoscopic disease activity for patients with long-standing UC, which can aid in determining individual treatment and follow-up strategies. For the first time, machine learning algorithms were introduced to UC endoscopic disease activity prediction. Moreover, the application of random forests, extreme gradient boosting, and synthetic minority oversampling technique algorithms had a good performance on the modeling. An interactive platform based on these models can be further developed with patients interacting conveniently in order to improve the database at the same time. Furthermore, it also will spur the development of digital health in this field.

Li, Tang et al. the authors applied to blood and stool biomarkers for variable screening through Logistic regression and a least absolute shrinkage and selection operator (Lasso) regression to construct a model for predicting the individual risk of moderate to severe endoscopic activity in UC patients based on four parameters or rather vitamin D, albumin, prealbumin, and fibrinogen with a concordance incidence of 0.860. This model can reliably predict the risk of moderate to severe endoscopic activity in UC patients through internal validation.

Interestingly, the model has the following advantages: decrease the frequency of colonoscopy and its potentials risks, decrease the cost of the examination and the burden of patients and, lastly, promote UC patient management in the local community thanks to the use of blood biomarkers in the primary hospitals.

Wang et al. identified cuproptosis-related genes (CRGs) and immune correlates with differential expression in normal and UC patient samples. Based on the expression of CRGs, UC patient samples were divided into two clusters and important immune-related differences between UC patients with different CRGs clusters were elucidated. Subsequently, the weighted gene co-expression network analysis algorithm was used to identify differentially expressed genes with enriched biological functions and pathways in both clusters. Finally, the authors constructed a machine learning model based on five CRGs (PLXDC1, WAS, CTSK, PLCE1, and LIMD2). Nomograms, calibration curves, decision curve analysis, and independent external datasets all validated the accuracy of the model. This study reveals the function of CRGs in UC for the first time, and the authors hope that the CRGs underlined in this study can provide important inspiration for subsequent studies on the functional mechanisms of UC guiding clinicians to make more individualized and precise treatment plans.

Lastly, Russo et al. in a systematic review and meta-analysis including 345 studies, reported that IBD alone does not seem to represent a risk factor for lymphoma development. Nevertheless, it is yet unknown whether IBD patients who have more severe and protracted disease activity are at higher risk than those who have less severe disease. Most of the body of evidence available in the literature has demonstrated that IBD patients receiving immunosuppressants such as azathioprine and mercaptopurines are at higher risk of developing lymphoma than the general population. A combined multidisciplinary management and a long-term follow-up are warranted in order to decrease mortality deriving from the coexistence of both conditions.

## Author contributions

VD'A: Writing—original draft. GG: Writing—review and editing.
